# Supplementary material for: All-at-once RNA folding with 3D motif prediction framed by evolutionary information
Source: Nat Methods. 2025 Oct 3;22(10):2094–106. doi: 10.1038/s41592-025-02833-w (PMC12510878; doi:10.1038/s41592-025-02833-w)
Supplement: Supplementary file 3 — Source code to run CaCoFold-R3D. Data and results. [file 41592_2025_2833_MOESM3_ESM.gz › supplemental_material/results/CaCoFold-R3D/CaCoFold-R3D_Rfam_vshuffle/RF02136_HTT-AS1_3.R2R.sto.pdf]

RF02136_HTT-AS1_3

5′- U ● U C C G U U G A G C C C C G C ● C C U U C G C C C R G G U G G G G C R C U G C G C U G U C A G ● G G Y Y U U G G Y G
